# A timeline of science and medicine in the technological age

**DOI:** 10.1590/acb415626

**Published:** 2026-10-05

**Authors:** Alberto Schanaider

**Affiliations:** 1 Universidade Federal do Rio de Janeiro – Faculty of Medicine – Department of Surgery – Rio de Janeiro (RJ) – Brazil.

**Keywords:** Technology, Generative Artificial Intelligence, History of Medicine, Science, Chronology

## Abstract

Coming up with a chronological definition of science and medicine is not an easy task. A terminological timely review seems to be necessary, especially considering recent advances in generative artificial intelligence. The present note suggests the introduction of a new period after the contemporary age to incorporate the development of deeply transformative technological innovation in modern science and medicine along the history of mankind.

Usually, definitions of the history of human civilization based on chronology are characterized by wars and revolutions. From an academic point of view, the ancient, middle, modern, and contemporary ages are the four well-known and classic periods of history. According to most European and North American scholars, the traditional contemporary age began nearly 230 years ago but has been radically disrupted over the last 80 years^1^.

It is no coincidence that the advent of great wars has marked the end of the first three ages. The twilight of the ancient age is related to the fall of the Roman Empire in 476 A.D.; and the middle age to the fall of Constantinople in 1453 A.D., and the Byzantine Empire, also referred to as the Eastern Roman Empire. The modern age ended with the fall of the *Ancien Régime* in 1789 A.D., and the French Revolution led the world into the contemporary age, which has continued to the present day^1^.

Keeping this chronology in mind, the present note proposes the use of a new scientific and historical timeline designation most suitable for our recent events, considering the exponential developments in all fields, in particular, medicine. In line with historical criteria separating previous ages, we propose to discontinue the contemporary age just after World War II in 1945 A.D., followed by the beginning of a new period, denominated as the technological age^1,2^ .

The atomic era in mid-1945, as well as the first programmable and fully operational computer built in early 1946 (the electronic numerical integrator and computer), represents key landmarks that identify this proposed new age of accelerated technological progress. Furthermore, the discovery of DNA in the 1950s should also be highlighted. Other remarkable scientific progress occurred, such as the launch of the first artificial satellite in 1957, the first man in space in 1960, and the first step on the moon in 1969^1,3^ .

Subsequently, it is noteworthy that in the last decades we saw outstanding advances such as robotic surgery, transplants and artificial or gene-modified organs, molecular biology with gene mapping and editing cell therapy (CRISPR and CAR-T), pharmacogenomics, individualized immunotherapy with checkpoint inhibitors, three-dimensional bioprinting and nanotechnological protheses, microchip implants, wearable devices, among others^3,4^ .

In 1974, the introduction of the internet democratized access to the frontier of knowledge, further blossoming a globalized and impactful technological transformation. Virtual agents and chatbots powered by advanced neural networks reached a new turning point in the early 2020s: generative artificial intelligence (AI). This emerging field brought the most disruptive digital technology ever known to science and medicine. AI-driven innovation has improved patients’ access to healthcare and the way in which medical records and examinations are processed and interpreted, speeding up diagnosis. Based on rigorous criteria, new AI systems also enable better patient monitoring and more precise, timely treatments, improving the overall quality of healthcare^3,4^ .

Nevertheless, we must emphasize that electronic health records, medical imaging data, including three-dimensional reconstruction, health tracking devices, telemedicine, among others, are complementary tools that assist medical professionals. In medicine, it is vital to keep in mind that these tools do not replace ethical or responsible human medical expertise, professional decision-making, or the demand for personal physician-patient relationships.

From chipped stone tools to the first quantum computer, every aspect of innovation has been driven by humanity’s ingenuity, and generative AI represents a bold step in science and medicine. We are living through the dawn of a new era: the technological age, spanning approximately the middle of the last century and the first quarter of the current one, leaving behind the contemporary age in the timeline of science and medical history.

## Data Availability

No datasets were generated or analyzed during the current study.
